# Supplementary figures and images for: Direct Salmonella injection into enteroid cells allows the study of host–pathogen interactions in the cytosol with high spatiotemporal resolution
Source: PLoS Biol. 2024 Apr 29;22(4):e3002597. doi: 10.1371/journal.pbio.3002597 (PMC11057982; doi:10.1371/journal.pbio.3002597)

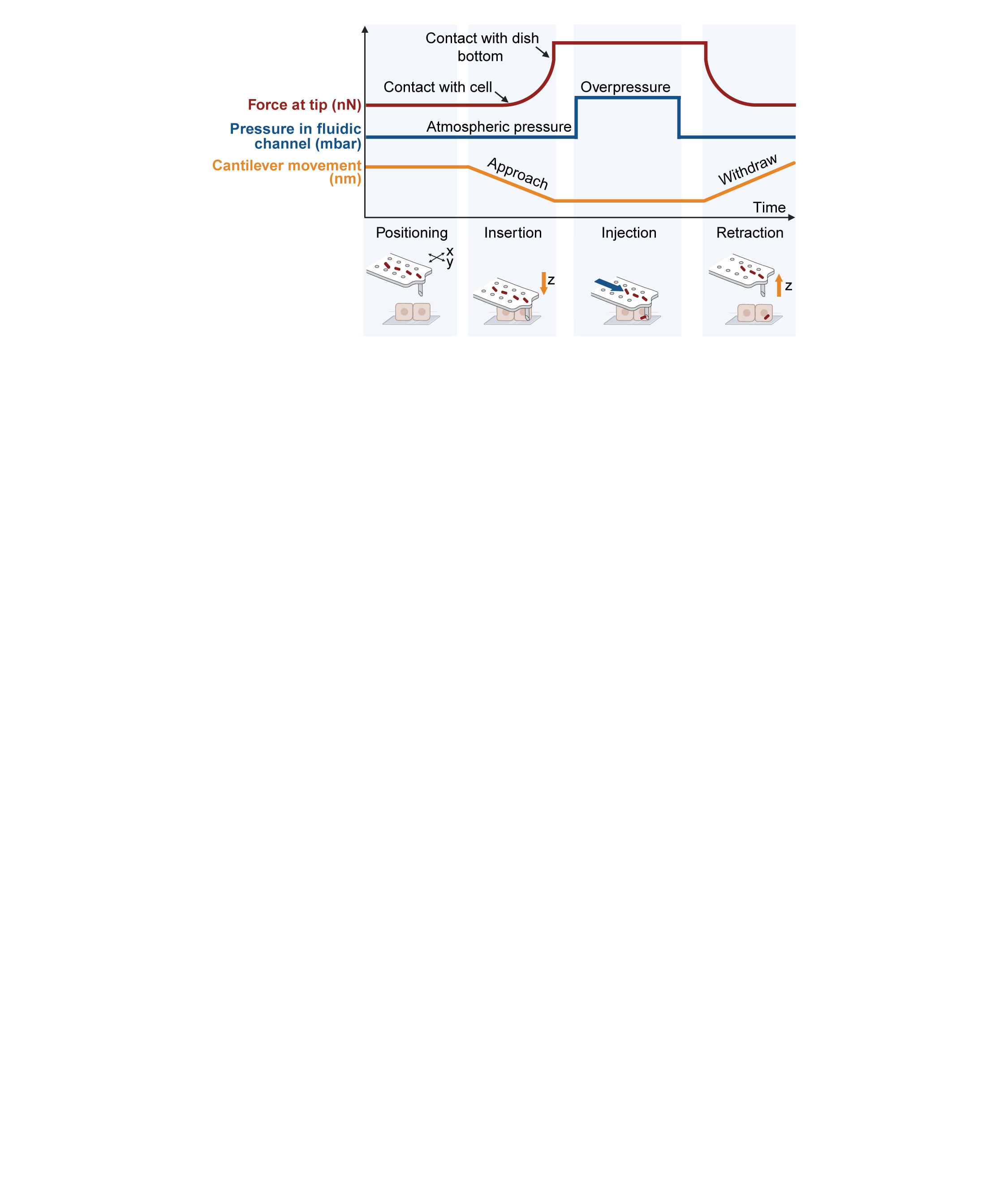

Supplement: S1 Fig — The FluidFM tip is positioned (x-/y-direction) above the target cell. Next, the tip is driven down (z-direction; yellow) into the cell until the dish surface is reached, a process during which the force of the tip is monitored (red) allowing for force-feedback control of the z-movement. When inserted, overpressure is applied within the microfluidics system (blue) leading to flow of the bacterial solution into the cell. To conclude the injection, pressure is returned to atmospheric level and the tip is retracted from the cell. Created with BioRender.com. (TIF) [file pbio.3002597.s001.tif]

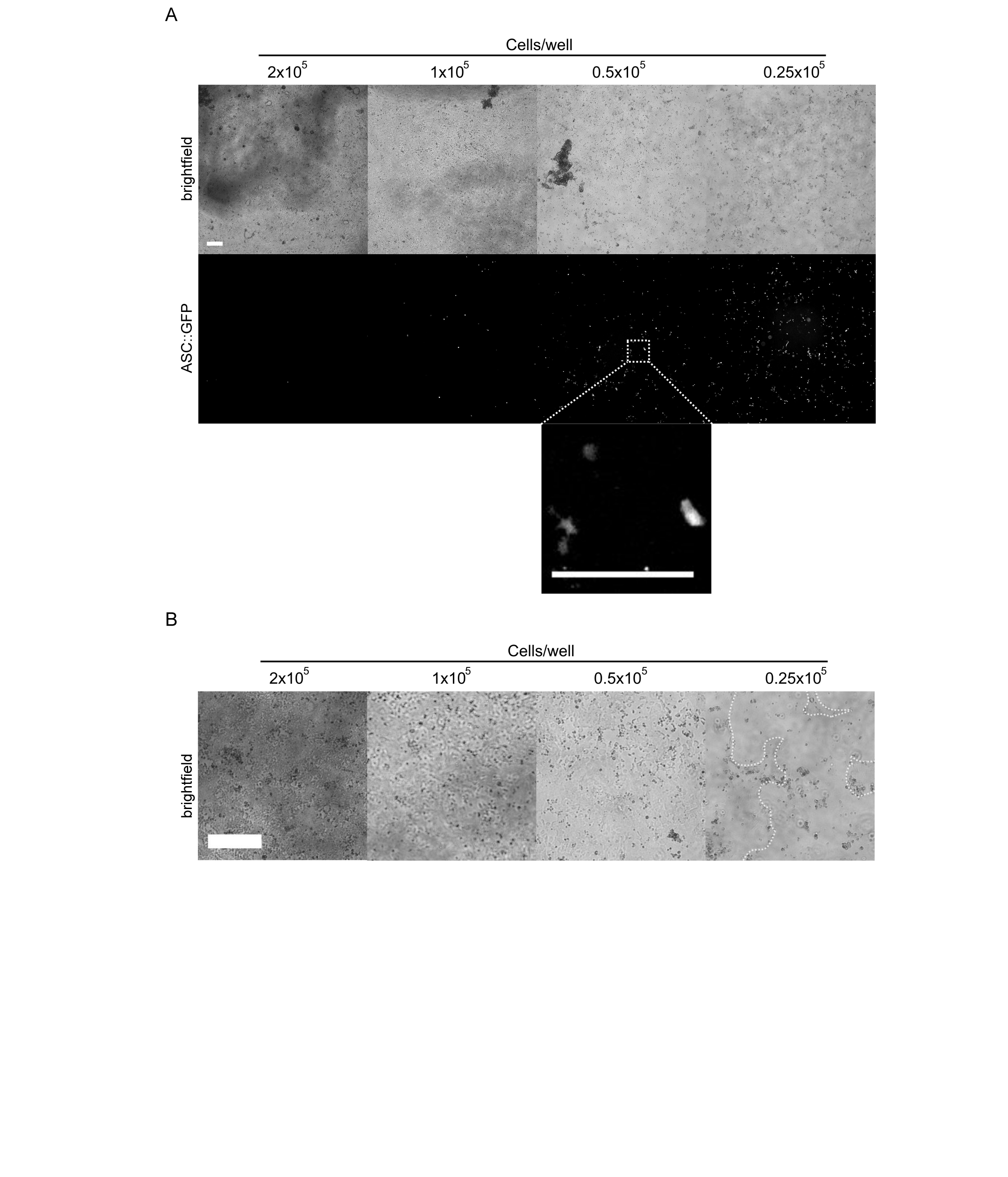

Supplement: S2 Fig — (A) Images acquired on day 3 after seeding of enteroid cells and transfecting with pSELECT-mASC-GFP, using Lipofectamine 3000. A zoomed image is depicted showing the cytosolic location of the ASC::GFP protein. (B) Cells at low densities are flattened with lower contrast and layers are less confluent. Dotted lines in 0.25 × 10^5^ cells/well indicate the border of the cell layer. Scale bars: 250 μm. (TIF) [file pbio.3002597.s002.tif]

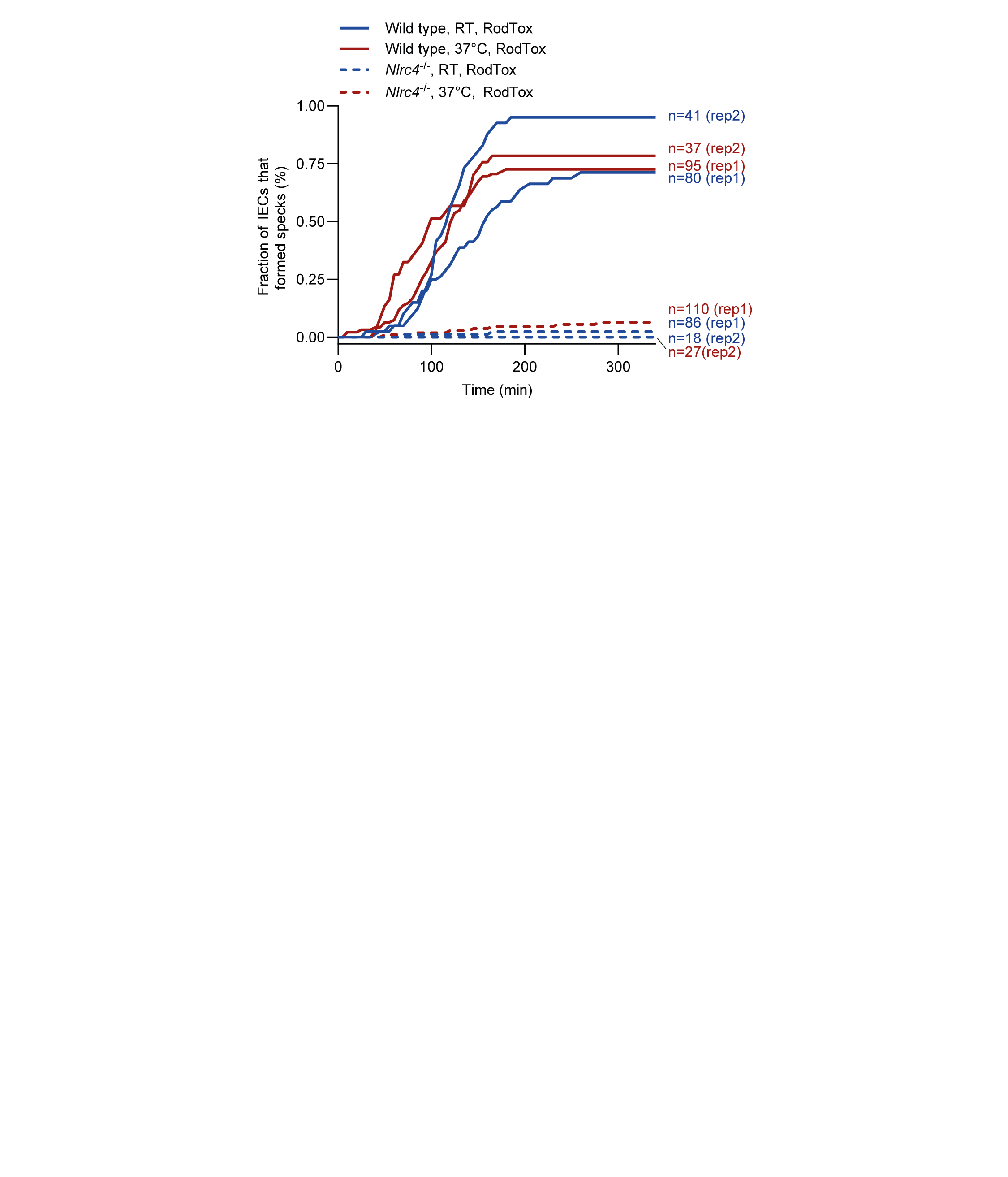

Supplement: S3 Fig — Cells transfected with pSELECT-mASC-GFP were treated with RodTox (LFNRod+PA) and the fraction of IECs reacting with speck formation at RT (blue) and 37°C (red) was quantified over time. Rep1 and rep2 indicate two biological replicas measured in two independent experiments. The data underlying S3 Fig can be found in S1 Data. (TIF) [file pbio.3002597.s003.tif]

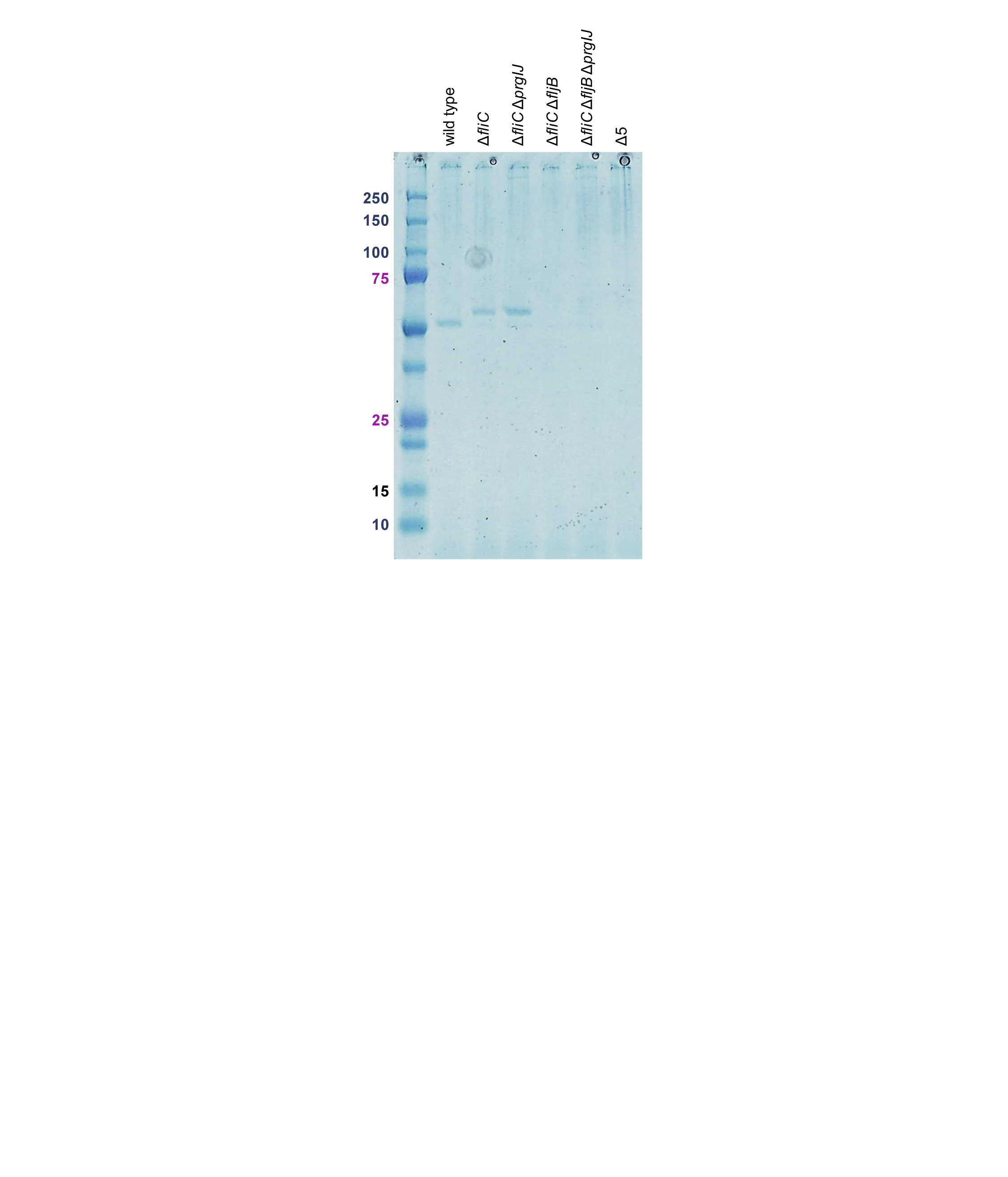

Supplement: S4 Fig — Supernatants of S. Tm cultures incubated overnight in LB were harvested by centrifugation, mixed with Lämmli buffer, and analysed by SDS-PAGE (mPAGE 8% Bis-Tris Precast Gel (Sigma-Aldrich), readyblue protein gel stain (Sigma-Aldrich), Precision Plus Protein Dual Color Standards (Bio-Rad)). The supernatants were loaded in an optical density-corrected manner to enable qualitative comparison of the detected protein. Culture supernatant of S. Tm wild type, S. Tm ΔfliC, S. Tm ΔfliCΔprgIJ, S. Tm ΔfliCΔfljB¸ S. Tm ΔfliCΔfljBΔprgIJ, and S. Tm Δ5 (ΔinvGΔsseDΔfliGHI) were analysed. Protein mass of the four NAIP/NLRC4 inflammasome-inducing ligands: FliC = 51.612 kDa; FljB = 52.536 kDa; PrgJ = 10.926 kDa; PrgI = 8.857 kDa. The raw image underlying S4 Fig can be found in S1 raw images. (TIF) [file pbio.3002597.s004.tif]

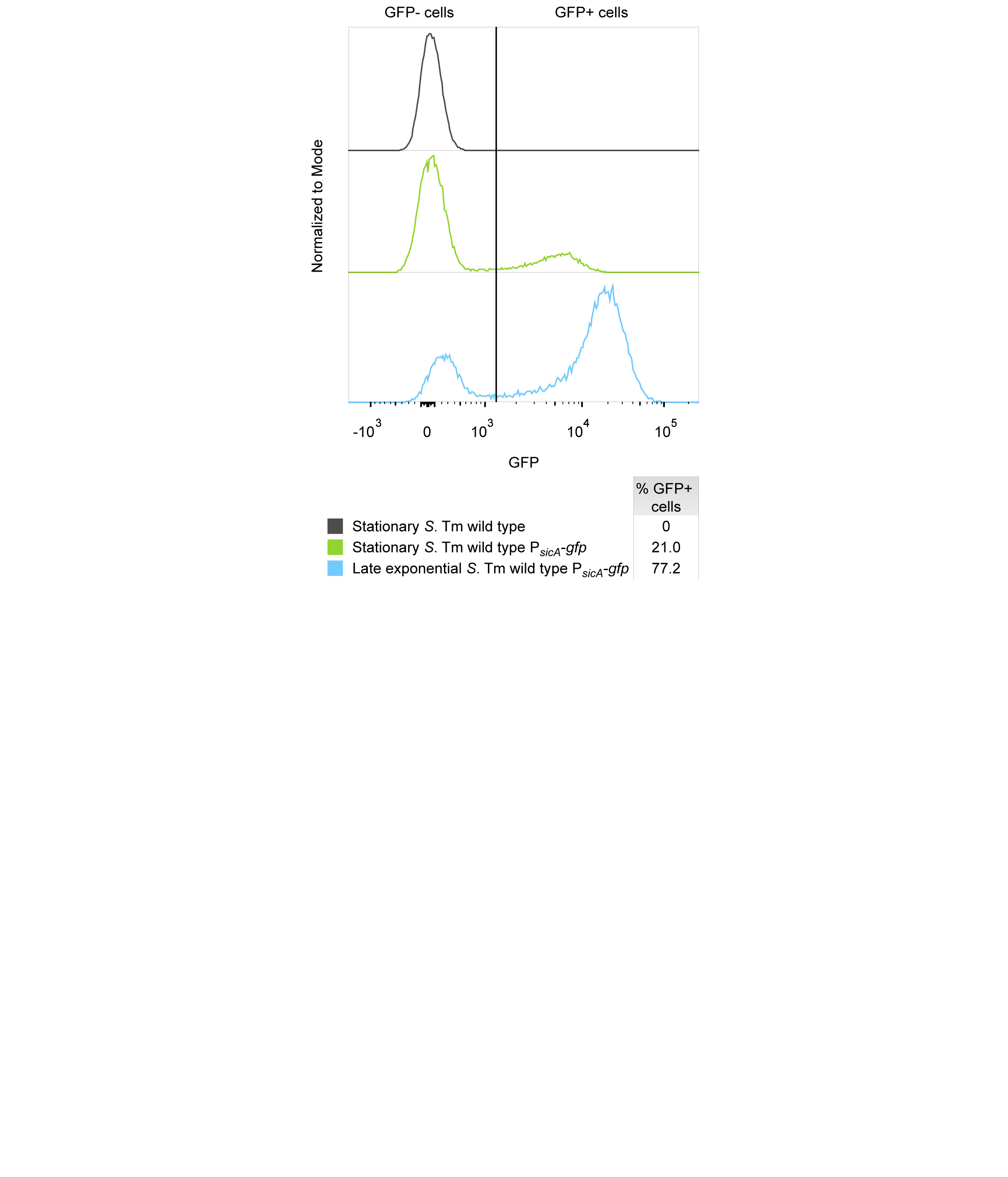

Supplement: S5 Fig — Bacteria were grown as for FluidFM injection experiments, i.e., to stationary phase via overnight incubation at 37°C in 10 ml LB supplemented with appropriate antibiotics in 100 ml baffled flasks (“stationary”). Additionally, bacteria grown to late exponential phase (1:50 diluted overnight culture, 4 h, 37°C) in LB with 0.3 M NaCl (final concentration) supplemented with appropriate antibiotics were analysed (“late exponential”). Bacteria were harvested by centrifugation, fixed in 4% paraformaldehyde (30 min, room temperature), washed 3 times in DPBS, and resuspended in DPBS. Bacteria were analysed using a flow cytometer FACSymphony A5 SE (BD Biosciences). The raw data underlying S5 Fig is available via https://doi.org/10.5281/zenodo.10829181. (TIF) [file pbio.3002597.s005.tif]

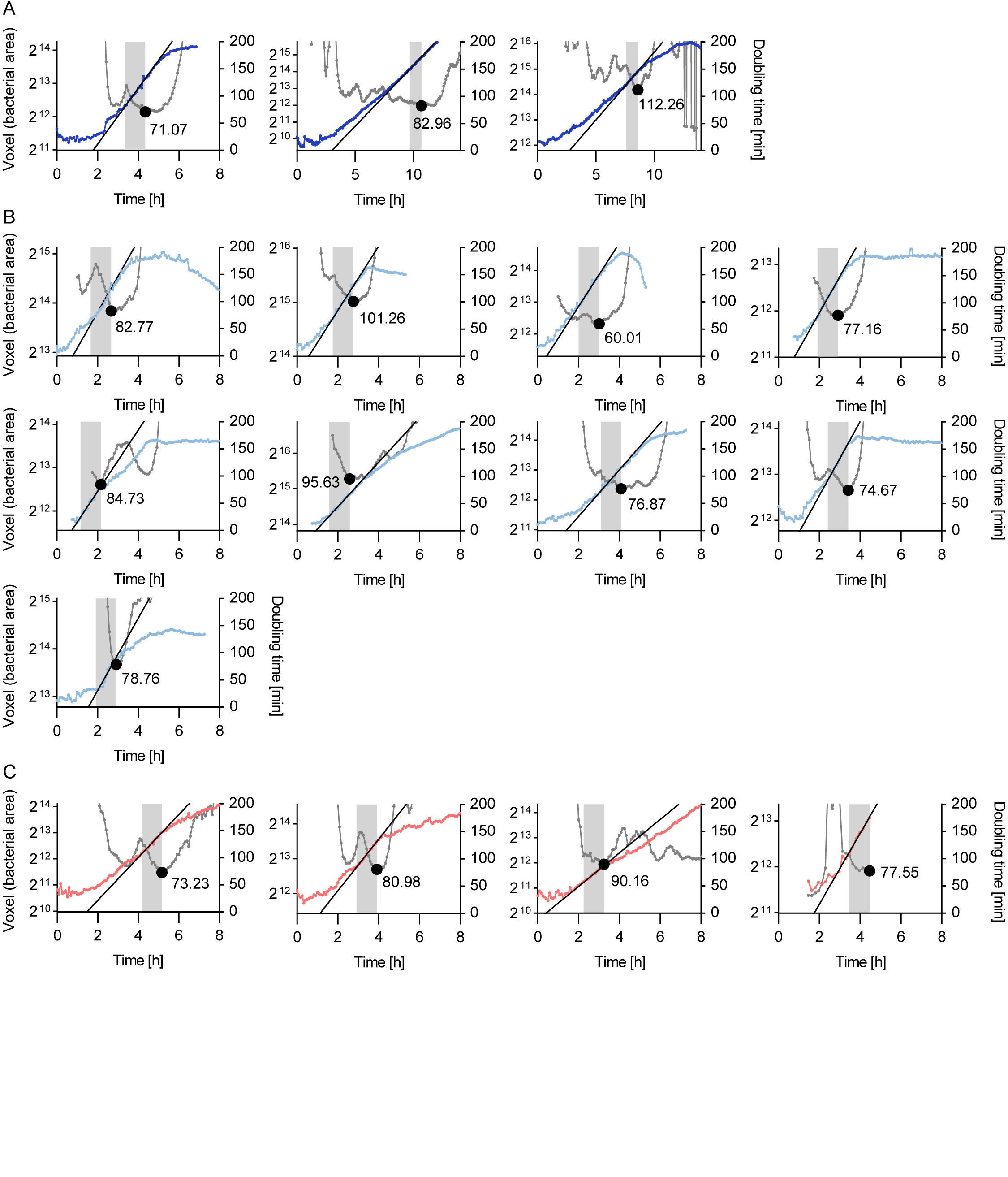

Supplement: S6 Fig — (A) S. Tm ΔfliCΔfljBΔprgIJ in wild type IECs (dark blue), (B) S. Tm Δ5 (ΔinvGΔsseDΔfliGHI) in wild type IECs (light blue), and (C) S. Tm wild type in Nlrc4-/- IECs (rose). Quantification of S. Tm FRFP fluorescent voxels over time in IECs depicted in colour, each plot showing growth of S. Tm in an individual IEC. Bacterial voxel doubling times were calculated over 60 min time intervals and plotted over time (grey dots). The minimal bacterial voxel doubling time (black dot with label (min)), the respective exponential fit (black line), and the respective 60-min interval (grey filled) are indicated. The same data as in Fig 4D and 4E. The data underlying S6 Fig can be found in S1 Data. (TIF) [file pbio.3002597.s006.tif]

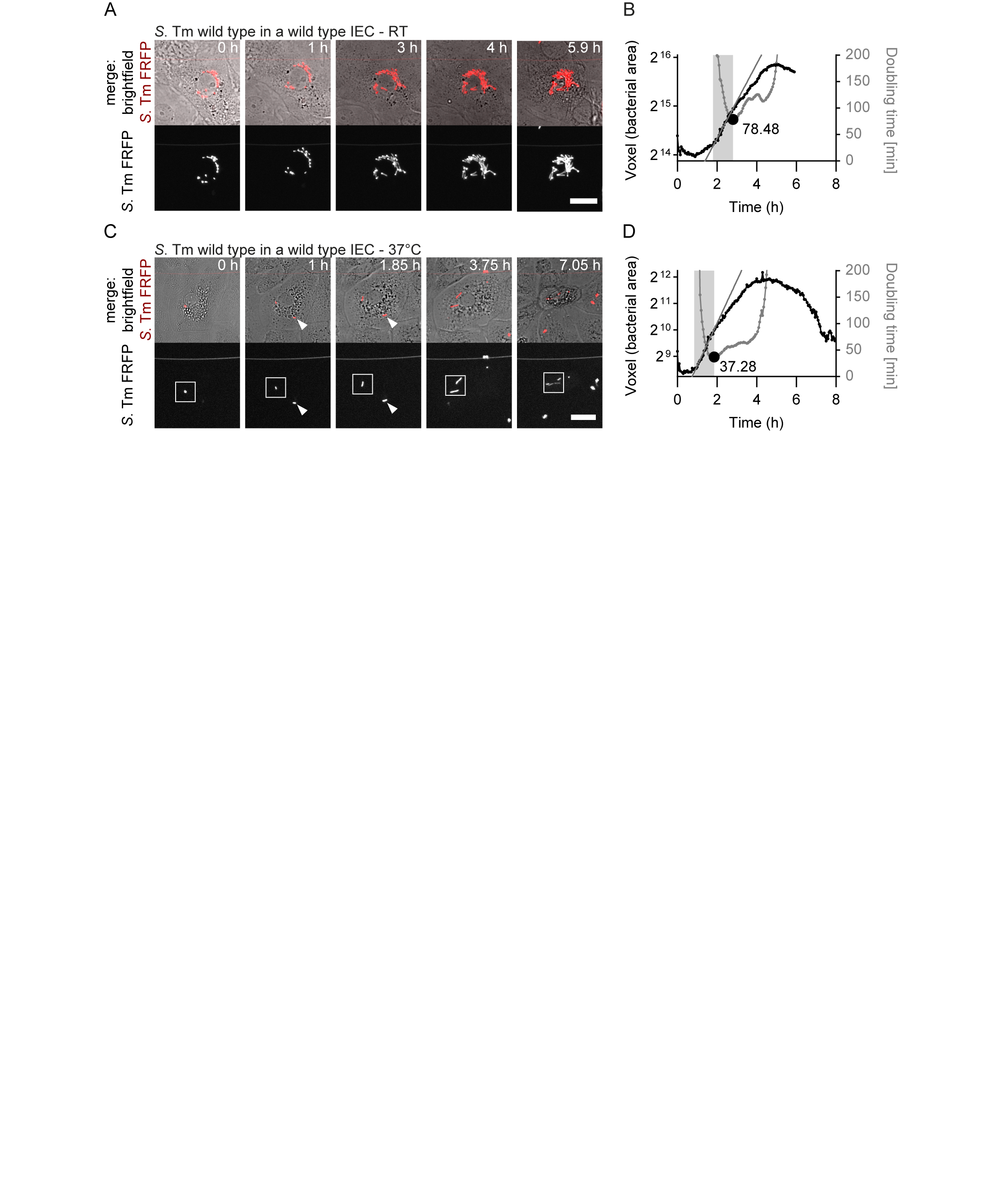

Supplement: S7 Fig — (A and C) Time lapse images of individual IECs injected with FRFP-labelled S. Tm. Injection and subsequent imaging were done at (A) room temperature (RT) and (C) at 37°C. To ensure that the observed growth is intracellular, gentamicin was added after injection. Scale bars: 20 μm. In C, the white arrow head indicates a non-intracellular bacterium that drifted away later in the time lapse and the white square indicates injected bacteria that are used for growth quantification in (D). (B and D) Quantification of S. Tm FRFP fluorescent voxels over time (black), showing the growth of S. Tm in the IEC in (A) and (C), respectively. Bacterial voxel doubling times were calculated over 60 min time intervals and plotted over time (grey dots). The minimal bacterial voxel doubling time (black dot with label (min)), the respective exponetial fit (grey line), and the respective 60-min interval (grey filled) are indicated. The data underlying S7B and S7D Fig can be found in S1 Data. (TIF) [file pbio.3002597.s007.tif]

Raw image of SDS-PAGE shown in Figure S4. Image acquired by sanning using a Nashuatec Printer.

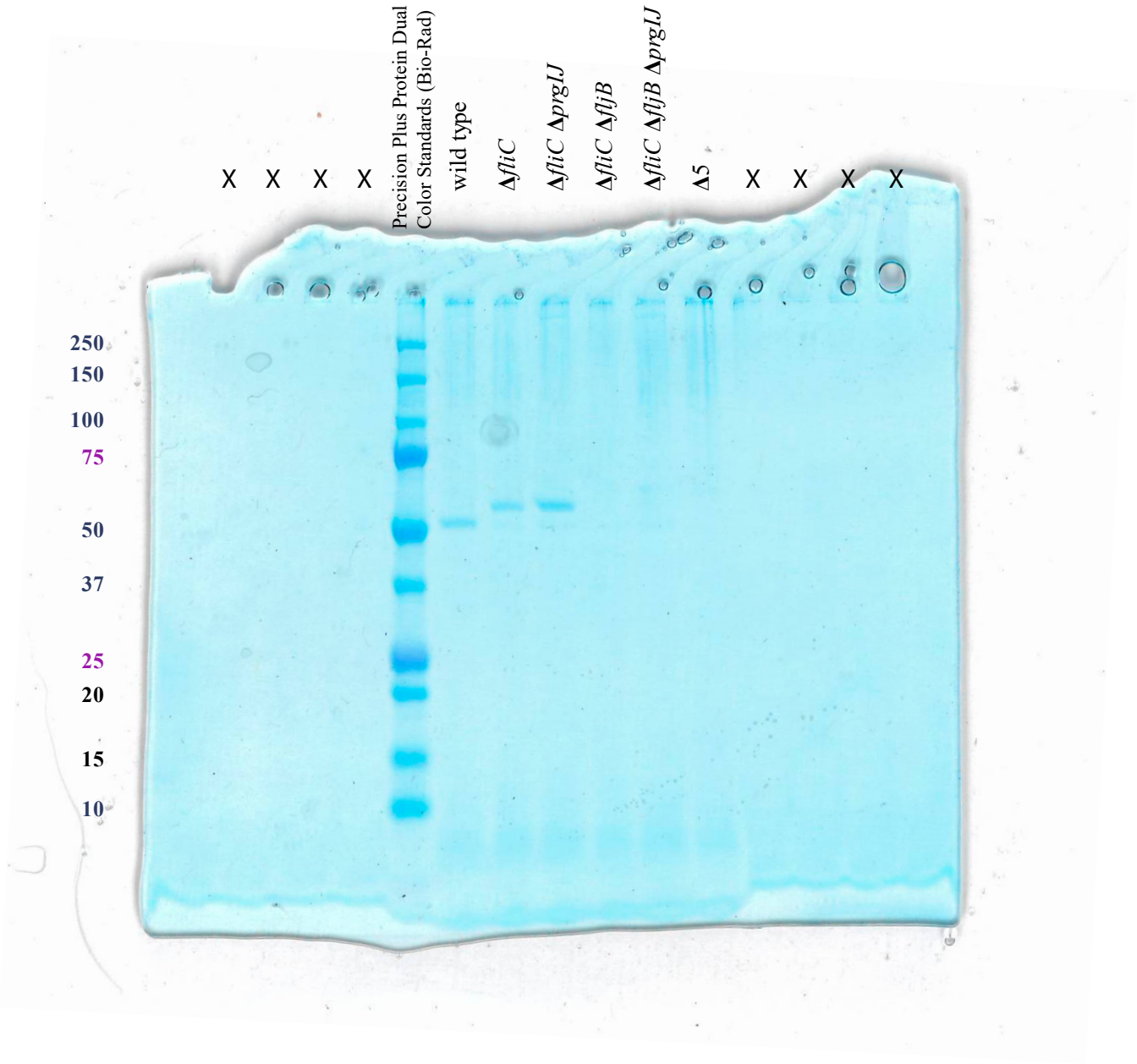

Supplement: S1 Raw images — (PDF) [file pbio.3002597.s009.pdf]
