# Supplementary material for: Direct Salmonella injection into enteroid cells allows the study of host–pathogen interactions in the cytosol with high spatiotemporal resolution
Source: PLoS Biol. 2024 Apr 29;22(4):e3002597. doi: 10.1371/journal.pbio.3002597 (PMC11057982; doi:10.1371/journal.pbio.3002597)
Supplement: S1 Table — (PDF) [file pbio.3002597.s010.pdf]

S1 Table Bacterial strains used in this study.

**Bacterial strain**	**Background**	**Relevant genotypes**	**Resistance**	**Source**
Salmonella enterica serovar Typhimurium (*S.* Tm)				
S. Tm wild type	SB300 (re-isolate of SL1344)	wild type	Sm	Hoiseth and Stocker, 1981 (1)
S. Tm ATCC 14028 Δ*fliC*	ATCC 14028	*fliC*::*cat*	Cm	Porwollik *et al*., 2014 (2)
S. Tm ATCC 14028 Δ*fljB*	ATCC 14028	*fljB*:: *kan*	Kan	Porwollik *et al*., 2014 (2)
S. Tm Δ*prgIJ*	SL1344	*prgIJ*:: *kan*	Kan	This study
S. Tm Δ*fliC*	SB300	*fliC*::*cat*	Sm, Cm	This study
S. Tm Δ*fliC*Δ*fljB*	SB300	*fliC*::cat, *fljB*::*kan*	Sm, Cm, Kan	This study
S. Tm Δ*fliC*Δ*prgIJ*	SB300	*fliC*::cat, *prgIJ*::*kan*	Sm, Cm, Kan	This study
S. Tm Δ*fliC*Δ*fljB*Δ*prgIJ*	SB300	Δ*fliC*, Δ*fljB*, *prgIJ*:: *kan*	Sm, Kan	This study
SB161	SB300	Δ*invG*	Sm	Kaniga *et al*., 1994 (3)
MvP101	ATCC 14028	*sseD*::*kan*	Kan	Medina *et al*., 1999 (4)
SB245	SB300	*sipABCD sptP*::*kan* *fliGHI*::Tn*10*	Sm, Kan, Tc	K. Kaniga and J. E. Galan
S. Tm Δ5	SB300	Δ*invG, sseD*:: *kan,* *fliGHI*::Tn*10*	Sm, Kan, Tc	This study

Abbreviations: Sm: streptomycin, Kan: kanamycin, Cm: chloramphenicol, Tc: tetracycline, *kan*: kanamycin resistance gene, *cat*: chloramphenicol resistance gene, Tn*10*: tetracycline resistance gene

References

1. Hoiseth SK, Stocker BA. Aromatic-dependent *Salmonella typhimurium* are non-virulent and effective as live vaccines. Nature. 1981;291(5812):238-9.
2. Porwollik S, Santiviago CA, Cheng P, Long F, Desai P, Fredlund J, et al. Defined single-gene and multi-gene deletion mutant collections in *Salmonella enterica* sv Typhimurium. PLoS One. 2014;9(7):e99820.
3. Kaniga K, Bossio JC, Galan JE. The *Salmonella typhimurium* invasion genes *invF* and *invG* encode homologues of the AraC and PulD family of proteins. Mol Microbiol. 1994;13(4):555-68.
4. Medina E, Paglia P, Nikolaus T, Muller A, Hensel M, Guzman CA. Pathogenicity island 2 mutants of *Salmonella typhimurium* are efficient carriers for heterologous antigens and enable modulation of immune responses. Infect Immun. 1999;67(3):1093-9.
