# Supplementary material for: Direct Salmonella injection into enteroid cells allows the study of host–pathogen interactions in the cytosol with high spatiotemporal resolution
Source: PLoS Biol. 2024 Apr 29;22(4):e3002597. doi: 10.1371/journal.pbio.3002597 (PMC11057982; doi:10.1371/journal.pbio.3002597)
Supplement: S2 Table — (PDF) [file pbio.3002597.s011.pdf]

S2 Table Plasmids used in this study.

Plasmids	Description	Construction	Source
pBAD-TDsmURFP-RBS-HO-1	Expression of TDsmURFP and H01 under control of pBAD, ColE1 ori, *amp*		Gift from Erik Rodriguez & Roger Tsien (Addgene plasmid # 80342) Rodriguez *et al*., 2016 (1)
pRJPaph-bjGFP	P_{aph}-*bjgfp*		Ledermann *et al*., 2015 (2)
pSEVA231	MCS, pBBR1, *kan*		Silva-Rocha *et al*., 2013 (3)
pSEVA331	MCS, pBBR1, *cat*		Silva-Rocha *et al*., 2013 (3)
pCE033	P_{aph}-*bjgfp*, pBBR1, *kan*	pRJPaph-bjGFP *Eco*RI/*Kpn*I (1083, 7627) into pSEVA231 *Eco*RI/*Kpn*I (16, 3107)	This study
pCE047	P_{aph}-TDsmURFP, ColE1 ori, *amp*	Gibson assembly of PCR product of pCE033 oCE0145/oCE0146 with pBAD-TDsmURFP-RBS-HO-1 *Bam*HI/*Nsi*I (4299, 1329)	This study
pCP20	FLP, repA101ts, *amp*, *cat*		Chereponov and Vackernagel, 1995 (4)
pKD46	Lambda red genes (γ, β, *exo*) under the control of P_{araB}, *araC*, repA101ts, *amp*		Datsenko and Wanner, 2000 (5)
pKD4	kanamycin resistance cassette and flp sites for KO via lambda red allelic exchange, *amp*, *kan*		Datsenko and Wanner, 2000 (5)
pSELECT-mASC-GFP	Expression vector encoding mouse ASC C-terminally fused via a six-amino-acid linker to GFP, *zeo*		InvivoGen (psetz-mascgfp)
pM972	P_{sicA}-*gfpmut2*, pR322, *amp*		Sturm *et al*., 2011 (6)
pCE051	P_{uhpT}-*gfpmut2*, pBBR1, *cat*	PCR amplified P_{uhpT} from the *S.* Tm genome TBA100/TBA105 *Xba*I/*Pst*I and pM972 *Pst*I/HindIII (752, 5418) into pSEVA331 *Hind*III/*Xba*I (24, 2944)	This study
pCE052	P_{aph}-TDsmURFP, P_{uhpT}-*gfpmut2*, ColE1 ori, *amp*	pCE051 *Sac*I/*Not*I (19, 945, 2901) into pCE047 *Sac*I/*Not*I (19, 4793)	This study

Abbreviations: *kan*: kanamycin resistance gene, *cat*: chloramphenicol resistance gene, *amp*: ampicillin resistance gene, *zeo*: zeomycin resistance gene

References

1. Rodriguez EA, Tran GN, Gross LA, Crisp JL, Shu X, Lin JY, Tsien RY. A far-red fluorescent protein evolved from a cyanobacterial phycobiliprotein. Nat Methods. 2016;13(9):763-9.
2. Ledermann R, Bartsch I, Remus-Emsermann MN, Vorholt JA, Fischer HM. Stable fluorescent and enzymatic tagging of *Bradyrhizobium diazoefficiens* to analyze host-plant infection and colonization. Mol Plant Microbe Interact. 2015;28(9):959-67.
3. Silva-Rocha R, Martinez-Garcia E, Calles B, Chavarria M, Arce-Rodriguez A, de Las Heras A, et al. The Standard European Vector Architecture (SEVA): a coherent platform for the analysis and deployment of complex prokaryotic phenotypes. Nucleic Acids Res. 2013;41(Database issue):D666-75.
4. Cherepanov PP, Wackernagel W. Gene disruption in *Escherichia coli*: TcR and KmR cassettes with the option of Flp-catalyzed excision of the antibiotic-resistance determinant. Gene. 1995;158(1):9-14.
5. Datsenko KA, Wanner BL. One-step inactivation of chromosomal genes in *Escherichia coli* K-12 using PCR products. Proc Natl Acad Sci USA. 2000;97(12):6640-5.
6. Sturm A, Heinemann M, Arnoldini M, Benecke A, Ackermann M, Benz M, et al. The cost of virulence: retarded growth of *Salmonella* Typhimurium cells expressing type III secretion system 1. PLoS Pathog. 2011;7(7):e1002143.
