# Supplementary material for: Direct Salmonella injection into enteroid cells allows the study of host–pathogen interactions in the cytosol with high spatiotemporal resolution
Source: PLoS Biol. 2024 Apr 29;22(4):e3002597. doi: 10.1371/journal.pbio.3002597 (PMC11057982; doi:10.1371/journal.pbio.3002597)
Supplement: S3 Table — (PDF) [file pbio.3002597.s012.pdf]

S3 Table Primers used in this study.

Primer	Sequence	Purpose
oCE0145	CGCGCGAAGGCGAAGCGGCATCT AGGGCGGCGGATTTGTC	Generation of pCE047 with Gibson assembly. Used on pCE033 together with oCE0146
oCE0146	CGTTGTTCAGAAGTTTTCATAATT ATATCTCCTTCTTATTTCTAGTCA AGATCT	Generation of pCE047 with Gibson assembly. Used on pCE033 together with oCE0145
oCE0154	GGGCGCTCATTTCAGTACGG	Verification/sequencing primer for *prgIJ* deletion, binds up stream of *prgI*. Used on *S*. Tm DNA together with oCE0155
oCE0155	AGCCTGTTCCTGGTCCAGTC	Verification/sequencing primer for *prgIJ* deletion, binds down stream of *prgJ*. Used on *S*. Tm DNA together with oCE0154
oCE0157	GGAAGTCATTATGGCAACACCTT GGTCAGGCTATCTGGATATATGA ATATCCTCCTTAGTTCC	Deletion of *prgIJ* via the lambda red recombination system using the PCR product from pKD4, used together with oCE0158
oCE0158	TATCGACGAATCATGAGCGTAAT AGCGTTTCAACAGCCCCTGTGTA GGCTGGAGCTGCTTC	Deletion of *prgIJ* via the lambda red recombination system using the PCR product from pKD4, used together with oCE0157
TBA100	ATACTCTAGACACGAAGCGTGAT ACACC	Generation of pCE051. Used together with TBA105 on the *S*. Tm genome to amplify P_{uhpT}.
TBA105	ATACCTGCAGGGATTACTCCTGA GCTAATTTTTATACC	Generation of pCE051. Used together with TBA100 on the *S*. Tm genome to amplify P_{uhpT}.
