# Supplementary material for: Direct Salmonella injection into enteroid cells allows the study of host–pathogen interactions in the cytosol with high spatiotemporal resolution
Source: PLoS Biol. 2024 Apr 29;22(4):e3002597. doi: 10.1371/journal.pbio.3002597 (PMC11057982; doi:10.1371/journal.pbio.3002597)
Supplement: Attachment — Submitted filename: 20240223_Response_To_Reviewers.pdf [file pbio.3002597.s013.pdf]

Reviewer #1: This manuscript introduces the use of Fluidic Force Microscopy to inject Salmonella Typhimurium directly into the cytosol of enteroid cells. This cutting-edge technique allows host responses to cytosolic Salmonella to be examined decoupled from invasion. The authors convincingly demonstrate that delivery of STM (or even STM media) is sufficient to induce NAIP/NLRC4/ASC inflammasome activation and pyroptosis. The experiments appear carefully conducted and documented. The authors are somewhat victims of their own success and the maturity of the Salmonella pathogenesis field in that these capabilities are incremental advances over their past exemplary work and the findings are in line with expectations based on past work. Still, the authors are able to draw conclusions with greater precision in timing and in examining cytosolic delivery in isolation from other host-pathogen processes. They also are able to draw the novel conclusion that replication in the cytosol does not require flagellin or either T3SS. They also note rare occurrences of WT STM growth within WT IECs—suggesting a rare population is able to escape induction of pyroptosis that could spur further work in the future.

Thank you for your positive assessment of our work.

The manuscript is well-written. The Introduction does an admirable job recapping the state of the field and the Discussion integrates their findings well with the literature and discusses future possibilities. A couple sentences in the Introduction contrasting FluidFM with more conventional microinjection techniques would be appreciated, and perhaps an additional paragraph in the Discussion to drive home what additional questions/discoveries can now be addressed by building off the technologies highlighted in this well-executed study. One possibility is examining genetic diversity across Salmonella serovars and/or from organoids derived from different inbred or collaborative cross mice using these same tools. Or perhaps comparing to human organoids with human-specific serovars.

Thank you, these suggestions help to highlight our technological advances for the general reader. We have expanded the introduction to include previous studies and findings on microinjection of bacteria into mammalian cells (see main text, Introduction). We agree, numerous applications will be possible in future studies of which we highlight a couple in our discussion, for example the study of effector proteins, autophagy, and host-microbe dynamics in general.

Reviewer #2: Summary
The authors develop an innovate way to combine microfluidics and atomic force microscopy to introduce Salmonella and bacterial products into the cytosol of intestinal epithelial cell cultures (2D organoids). They find that bacterial activation of NAIP/NLRC4 inflammasomes by flagella or T3SS rod components induces rapid cell death. Concomitantly, bacterial growth is limited in the cytosol of the cells. The paper provides an exciting new tool to study bacterial colonization of the cytosol, and the defenses that protect this compartment. The tool will be applicable to the study of other pathogens/chemical entities. I am in favour of publication with only a few comments for the authors consideration.

Thank you for your positive assessment of our work.

Major comments

-Prior studies of bacterial growth in the cytosol of cell lines should be discussed, particularly where microinjection of bacteria was performed (PMID).

We have expanded the introduction to include previous studies and findings on the cytosolic growth of bacteria upon microinjection into mammalian cells using immortalized/transformed cell lines (see main text, Introduction).

-Figure 2A. Is the injected dye really bleaching? Or leaking out of the cell? Perhaps an external dye (e.g. PI) could be added to test this alternative possibility.

Indeed, we could not definitively assert that the dye undergoes bleaching based on the presented data, although leaking would likely have led to cell death. To demonstrate that the membranes of injected cells do not exhibit leakage following injection, we conducted an additional control experiment. We introduced the dye (CF640R hydrazide) into ASC::GFP-labelled IECs and monitored whether the GFP signal diminishes over time, which would suggest membrane leakage. We observed that although the fluorescence intensity of CF640R decreased over time, the GFP signal remained optically unchanged and the cell remained viable and within the monolayer, both indicators for the preservation of membrane integrity (see Figure below). We have adjusted the description of the legend for Figure 2 and deleted the statement "time lapse imaging revealed bleaching". It now reads "While dye intensity decreased over time, probably due to quenching or bleaching, time lapse brightfield images …"
Even though, other dyes, such as lucifer yellow (which has been used for FluidFM injections before, e.g. Guillaume-Gentil *et al*. 2012), might have been visible for a longer period of time after intracellular delivery, we chose to use this far-red dye in the current study, firstly to keep imaging gentle using the long-wavelength laser, and secondly to maintain similar imaging conditions compared to bacteria injections in later experiments, which are labelled with a far-red fluorescent protein.

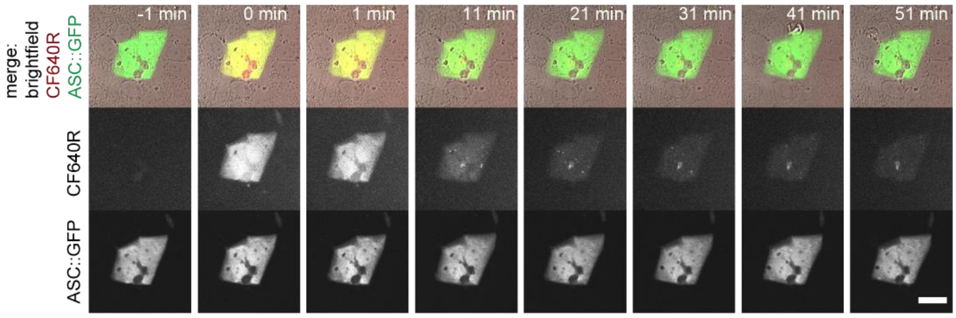

Figure: FluidFM-mediated injection of far-red fluorescent tracer dye (CF640R hydrazide; f.c. 0.1 mg/mL in HEPES-2) into a ASC::GFP expressing wild type enteroid-derived IEC. Microscopy images showing the cell prior injection (-1 min), directly post injection (0 min) and a time lapse with 10 min intervals (1 min and following). Scale bar: 20 µm.

-Figure 2A. How many cells were tested with this control? Was there no cell death ever? Since this is a new technique it is important to document the success rate.

This comment is addressed together with the one below.

-Same comment for Figure 2B-D, quantification is required. Perhaps the same way data is represented in Figure 3C but with the observed phenotypes plotted over time: contrast of nucleus, cell shrinkage, blabbing, explosion, etc. Caspase inhibitor could be included. This would highlight the power of the ASC spots as an assay of cell death, since the timing seems to be very fast.

The success rate of FluidFM-mediated injections has been thoroughly evaluated by Guillaume-Gentil *et al.* 2014 (pyramidical FluidFM tips: 100% for nuclear dye injection) and by Gäbelein *et al*. 2022 (cylindrical FluidFM tips as used in this study: 95% for mitochondria transplantation).
While "success" is defined as a successfully injected cell and its subsequent survival, this readout becomes inherently problematic in our study, where cell death/pyroptosis upon injection is studied. As pointed out by the reviewer, the ASC::GFP as a life-cell reporter was therefore a powerful tool to obtain quantitative data based on a more specific phenotype in living cells instead of relying on the phenotype "death". To avoid a misunderstanding about the goals of the experiment shown in Figure 2, we have added "to obtain phenotypic data on cell fate" (see main text, results).

-pg6. I appreciate that the authors include a rare occurrence where WT bacterial growth is normal within WT cells. This could be important for future studies of IECs. However, I think it is a bit jarring and misleading to include it in Figure 5D/F. I think the readers should see the true growth of WT bacteria in WT cells,.. from Figure 2B it seems there is some growth prior to cell death, which would be expected in the time scale studied. Ideally Figure 5F would have a plot of all growth rates for WT bacteria in WT cells (which would be mostly low), including the outliers that look normal.

We agree that the inclusion of rare occurrences of WT bacteria in WT cell growth in Figure 5 might be confusing for the reader.

However, incorporating "non-growth" data poses challenges for several reasons:

- Bacteria usually exhibit a lag phase of 1-2 h after injection before growth is observed (as seen in Figure 5D), a period during which immunocompetent cells often undergo cell death.
- Further, non-growth could mean i) host cell death due to injection, ii) host cell death due to host responses, or iii) bacteria released extracellularly, in which cases bacteria are inhibited in growth due to gentamicin exposure, or iv) non-viable bacteria.

Considering these factors, we think that including "non-growth growth curves" would add complexity and potentially ambiguity to the data presented.

Instead, we address the concern by showing the rare cases as a supplementary figure (S6), and show the representative cases in the main figure.

Also, we added the fluorescent images of the bacteria to Figure 2B, to show that within the timeframe depicted (injection to cell death - one hour post injection) there is no bacterial growth.

The bacteria merely dissociate from the injection site, which could in the merged image be mistaken as growth.

Minor comments
Pg4, line 152 "Rodtoxone"… add space
Corrected, see main text.

Pg8, line 295 "high almost wild type", a comma after "high" might make the sentence flow better
Adopted, see main text.

Reviewer #3: In this short report Ernst et al, have used Fluidic force microscopy to deliver Salmonella Typhimurium directly into the cytosol of cultured murine enteroid cells. This approach allows them to uncouple cytosolic events from attachment, invasion, and vacuolar escape. They then confirm that, as previously established by several laboratories, Salmonella virulence factors, specifically the T3SS1 and flagellar proteins, stimulate caspase-1 dependent cell death. Although the study does not provide any major insight into STm pathogenesis, the method described has great potential in the current "single cell" era. Overall, the paper is well written, and the experiments are technically sound, however, there are some issues that should be addressed.

Thank you for your positive assessment of our work.

Major Concerns

1. The experiments have been done at two different temperatures (37°C and room temp). The mammalian cells are initially maintained at 37°C (physiological) until the bacteria are injected. Thereafter, since the bacteria in the FluidFM system are at rt, the authors made the decision to continue at rt. However, both mammalian cells and bacterial cells behave differently at rt vs 37°C. Membrane fluidity, cell viability, motility, and growth rate can all be impacted. For example, in HL-60 cells incubation at rt has been shown to lead to caspase-1 dependent cell death (FEBS Lett. 1997 Nov 17;417(3):379-84. doi: 10.1016/s0014-5793(97)01327-6) and replication of Salmonella at room temp would likely be reduced compared to 37°C. The only experiment that was maintained at 37°C throughout, was the one in which the authors tested the requirement for caspase-1 in cell death (Fig 2D). However, given the experimental design, it possible that the observed decrease in cell death was due to the incubation temperature (37°C) rather than, or in addition to, the caspase 1 inhibitor. Appropriate controls should be included or the authors must clearly state and discuss that results from cells at rt cannot be directly compared to those from experiments done at 37°C (especially rate of bacterial replication and cell death). As presented, this (non-physiological temperature) appears to be a major limitation to the approach.

We agree this is a valid point. We repeated the injection of *S*. Tm into Caspase-1 inhibitor treated cells at room temperature and updated the corresponding Figure panel (Figure 2D). The results were consistent with the previous findings at 37°C: The shown injected cell remains viable for multiple hours, and bacterial growth can be observed.

Regarding the earlier study Shimura et al., 1997, FEBS Letters. The authors indeed showed that incubating the human promyeloblast cell line HL-60 cells at room temperature induces cell death that can be prevented with a caspase-1 inhibitor. However, they also note that Caspase-1 is not cleaved to its active form at room temperature, suggesting that a Caspase-1-like protein is responsible for the RT induced cell death. Besides this cell line, the authors also investigated RT-induced apoptosis in more than 10 other human cell lines and found that only one, a T-cell lineage, showed a similar phenotype, suggesting that most cell lines do not increase apoptosis at room temperature.

Thus, apoptosis at room temperature seems to be a rather rare effect, and dependent on the cellular model used.

We investigated the impact of temperature on the host reponse investigated here, i.e. ASC speck formation as an indicator for inflammasome activation in cells from the enteroid monolayers, the specific cellular model used in our study. We observed similar frequencies of ASC speck formation upon RodTox treatment regardless of the temperature at which the assay was conducted, i.e. room temperature vs. 37°C. We therefore conclude that ASC speck formation in enteroid monolayers does not appear to be affected by the chosen temperature in terms of the observed biological outcome. We have included the newly generated data on temperature control in a dedicated figure in the Supplementary Material of the revised manuscript (Figure S2).

Regarding the impact of temperature on bacterial replication, please see reply to point 2.

2. It would be helpful to do a direct comparison of T3SS1-mediated uptake vs FluidRM injection. Specifically, is the rate of intracytosolic bacterial replication the same in both cases? Is this dependent on temp?

We agree, this is an interesting point. However, obtaining equivalent high-resolution data on intracytosolic replication post-invasion, akin to what we can achieve with FluidFM injected bacteria, poses significant challenges. Firstly, only a small fraction of bacteria manages to escape into the cytosol following invasion, rendering it difficult to pinpoint which cell to image with high resolution. Additionally, the timing of cytosolic entry is not precisely known, and the diverse locations of bacteria (adhering, invading, vacuolar, cytosolic) further complicate the analysis of a singular population.
In our view, this showcases the novelty and power of the FluidFM injection approach, which reaches high temporal and spatial resolution.

Regarding the temperature dependence of bacterial growth. We record cytosolic *S*. Tm voxel doubling times of about 80 minutes at our experimental conditions, thus at room temperature (Figure 5). In a separate experiment, we achieved to record the growth of *S*. Tm in an IEC at 37°C (Figure S6, one of the rare events of wild type bacterial growth in a wild type IEC). The calculated minimal voxel doubling time was 37 min, which is consistent with the doubling time reported by Gäbelein *et al*. 2022, where *Salmonella* injected into HeLa cells at 37 °C had a cytosolic doubling time of 35 ± 5 min. We adapted the result section (see main text) to point the reader to Figure S6, where this one case of cytosolic growth of *S*. Tm at 37°C can be found.

3. What percentage of the STm injected into the host cells are expressing T3SS1? SPI1 expression is bistable, with SPI1-OFF and SPI1-ON populations co-existing even when STm are grown under SPI1-inducing conditions. Whereas T3SS1-mediated invasion selects for T3SS1-induced bacteria getting into the host cell this is not the case for FluidFM injected bacteria. It's important to have some information on what % of injected bacteria are T3SS1-induced and can be done using a fluorescent reporter to differentiate between SPI1-OFF and SPI1-ON bacteria both in the "inoculum" and following injection into the cytosol. I consider this essential for the study.

To address this comment, we assessed the fraction of T3SS-1-induced bacteria in a culture of *S*. Tm grown under the described experimental conditions. We utilized the fluorescence-based T3SS-1 reporter P_{sicA}-*gfp* (pM972, Sturm et al., 2011, PLoS Pathog) and analysed the bacterial population by flow cytometry. Approximately 20% of the population was GFP positive, which confirms the presence of a SPI1-ON population. We included these data to the revised manuscript and additional controls, i.e. previously described “SPI-1-inducing conditions” (approx. 80% GFP positive) in Figure S4 of the revised manuscript.

4. Are the bacteria injected directly into the cytosol able to respond to that environment in the same way as they would following T3SS1-mediated invasion. One way to show this would be to use a fluorescent reporter e.g. under the control of the uhpT promoter, to show whether the bacteria are respond to the cytosolic environment.

We agree, this is a valuable control to show that bacteria respond similarly to the cytosolic environment. We now tested the widely used uhpT promoter (P_{uhpT}-*gfp*), which is induced upon glucose-6-phosphate exposure of the bacteria, and included the data to the manuscript (Figure 5, panels F and G). The GFP intensity of FluidFM-injected *S*. Tm P_{uhpT}-*gfp* visibly increases at the onset of bacterial growth within the host cell (Figure 5F). Further, we determined the mean GFP intensity of

the bacterial voxels over time and report that, in contrast to the constitutively expressed far-red fluorescence protein (FRFP), GFP intensity increases.
These data allowed us to conclude that injected bacteria are able to respond to the cytosolic environment in similar ways as described for *S*. Tm that reach this niche by T3SS1-mediated invasion.

Other comments
1. The bacterial supernatant was highly concentrated compared to the bacterial injections and this should be noted in the results section.

Addressed, see main text result section.

2. Line 327- ref 61 should be included here for SopB induction of Akt

Our understanding is that the study of Chong *et al*., 2019, Mol Microbiol focuses on the effector SipA rather than on SopB and its induction of Akt. We thus find the reference misleading in the context described at this specific point of the manuscript.

3. Line 359-361 - there are some typos.
Corrected three typos in these lines (twice form to from, once S. Tm to *S*. Tm).

Reviewer #4: This manuscript uses a previously developed bacterial-delivery approach to target Salmonella directly to the cytosol of host cells. The authors use this for the cytosolic delivery into enteroids, a physiologically relevant model for intestinal infection of this pathogen. Through this method, they bypass the natural cellular targeting, entry, trafficking and vacuolar escape. Therefore, they are able to test bacterial mutants that play a role in upstream processes as well as events triggered within the cellular cytosol. This is very elegant. Through this method, the authors could confirm in this pertinent model the major role of the NLRC4 inflammasome triggered through flagella and T3SS components in restricting cytosolic growth. Interestingly, the phenotype presented some leakage for bacterial growth in some cells indicating that there is cell-to-cell variability that may allow escape from growth restriction in a the cytosol of a few cells. Unfortunately, the approach does not seem to have enough throughput to obtain more robust data on this cell-cell variability. Nevertheless, there are a number of excellent novel elements in this manuscript: first, the generation of the ASC-reporter in mouse intestinal enterocytes will be an interesting tool for numerous researchers. Secondly, the clearcut analysis of the important implication of NLRC4 in Salmonella growth restriction (compared with other pathways) is an important information to drive future research. I feel that a better characterization (eg through purified flagella components), and also more controls on the amount on initially delivered pathogens (it's not clear to me how many bacteria are "injected" in each cell) would be important to make this work well rounded and interesting for a large audience- please see below. The manuscript is very carefully written and analyzed reflecting the top level research of the involved labs.

Thank you for your positive assessment of our work.

Specific points:
Cytosolic delivery of intracellular bacteria avoiding the natural entry route has been a recurrent theme of infection biologists, and this has been done for more than 20 years by micro-injection. The FluidFM method is another approach for this, and I would have appreciated some benchmarking, especially as the Goebel lab has already performed similar experiments (not on Salmonella though) more than 20 years ago and has defined differential cytosolic growth depending on the kind of injected pathogen (they focused mainly on Listeria at the time). It's a bit of a shame that such work is not cited nor discussed at all, or that relevant controls with other pathogens are shown.

As suggested, we have expanded the introduction to include previous studies and findings on microinjection of bacteria into mammalian cells (see main text, Introduction).

The supernatants from wildtype bacteria have the strongest effect in the induction of the specks and thus triggering bacterial restriction. It is still not absolutely clear how different factors play together in NLRC4 activation. Experiments I would recommend: 1: check purified flagellin components in the model of the manuscript and their impact in restriction. 2: Check whether the strong induction through the WT supernatants still takes place when added to the different bacterial mutants that do not induce the specks. 3: Make dilutions of the supernatants, and take supernatants from different time points of bacterial growth (exponential phase versus stationary phase) and check the extend of speck induction.

We agree that an investigation of dosage effects and pure PAMPs (such as flagellin) or ligands on inflammasome activation will be interesting. Our present study lays the groundwork to address this point in the future. However, we believe that a thorough investigation is beyond the scope of this publication and should be the focus of a dedicated follow-up study. Note, it needs a rather high number of dedicated injections to generate quantitative data to study concentration effects and these need to be coupled each to “long-term” observations that cannot be parallelized (at least not currently with the present setup).

The authors need to provide experimental controls on the precise bacterial numbers initially delivered to get more robust information on how bacteria induce the NLRC4 pathway. As it is likely that the inducers are shed from the cytosolic bacteria, there is an enormous difference if initially 1, 2, 3… bacteria are injected. This is needed to fully understand the evasion of restriction in some cells.

This point addresses the dose of the inducing ligands in our experiments. Interestingly, we were able to show that it is not necessary to deliver bacteria, but that the surrounding buffer is sufficient to induce a host response. Regarding the evasion, we agree that exposing the host cell to less ligands can be an evasion strategy. However, in the one case where we observed growth of wild type bacteria in wild type cells, this was unlikely because more than 10 bacteria were injected (Figure S6A). As mentioned above, we agree that investigations of the dose of ligands and the respective host response are future directions that should be studied.

Also, along these thoughts- the authors should discuss how the entry through the bacterial containing vacuole may affect the amount of NLRC4 inducers compared to the used bacteria- I would guess that a single bacterium delivered as in the manuscript will have much more flagella components attached to it than a bacterium that has traveled through a Salmonella containing vacuole. Perhaps the authors can check this even experimentally?

Indeed, bacteria that are residing within a vacuole downregulate the NRLC4 ligands, i.e. flagella and the T3SS-1, as we mention in the introduction. Thus, it is conceivable that after vacuolar egress *S*. Tm harbour less of these compounds. We report on the induction potential of specific ligand knock outs strains of *Salmonella*, which may mimic, at least to some extent, bacteria that down regulate these components.
Interesting to note: cytosolically growing *S*. Tm were described to upregulate the ligand containing structures again (see introduction), which adds another layer of complexity to this question.

I was puzzled that the growth experiments of cytosolic bacteria seem to have been done at room temperature (perhaps I misread?). This is physiologically problematic as the expression and regulation/action of the factors involved in triggering ASC are temperature-dependent, and a higher temperature (30 to 37 degrees) seems to be more appropriate. Otherwise, it is difficult to fully understand the experiments.

We have now conducted control experiments that validated that ASC induction also occurs at the temperature of our experiments. We refer the reviewer to our response to comment 1 and 2 of reviewer #3.

Do the injected bacteria secrete bacterial effectors? Normally T3SS is activated through the entry process and afterwards (for T3SS-2). The injected effectors during this process may further modulate the NLRC4 pathways, and in case T3SS are not efficiently activated, it is complicated to understand the phenotypes. I suggest to add as a control the injection of bacterial strains that are constitutively secreting effectors and check the impact on speck formation and bacterial growth.

We agree that the technology presented here would be an ideal platform to address questions regarding effectors, especially those that impact host cell responses. In our view, however, this is out of the scope of the current manuscript.

We address the relevance of effectors in the discussion and regard it as outlook of the study: “it is also conceivable that the pathogen might be able to suppress this response, e.g. via some uncharacterized T3SS effector proteins.” … “Notably, *S*. Tm does possess T3SS effectors that affect host cell death pathways, such as SopB or SopF influencing the Akt survival pathway or caspase-8, respectively ([Knodler *et al.* 2005, Yuan *et al.* 2023]). However, no effectors are currently known to directly modulate the NAIP/NLRC4 inflammasome machinery. FluidFM-enabled cytosolic access of

host cells could provide a valuable platform to further investigate effector-driven manipulations of host defense responses employed by *S*. Tm."

I think Fig3 should be rather a supplement as it cuts the flow of the work- it's simply testing whether the reporters work in the model of the manuscript.

We understand the concern that the paragraph cuts the flow of the result section and we have considered to move Figure 3 to the Supplements. However, there is important information in the corresponding paragraph describing this figure that is important to understand the data in the following figures, i.e. that a low fraction of IECs do not form specks and thus might allow for the rare cases of wild type bacteria growth, and that there is some background activation of specks due to high ASC::GFP expression.
Therefore, we prefer to keep Figure 3 as a main figure to make the following data more easily understandable.
